# NAD Metabolism in Cancer Therapeutics

**DOI:** 10.3389/fonc.2018.00622

**Published:** 2018-12-12

**Authors:** Keisuke Yaku, Keisuke Okabe, Keisuke Hikosaka, Takashi Nakagawa

**Affiliations:** ^1^Department of Metabolism and Nutrition, Graduate School of Medicine and Pharmaceutical Science for Research, University of Toyama, Toyama, Japan; ^2^First Department of Internal Medicine, Graduate School of Medicine and Pharmaceutical Science for Research, University of Toyama, Toyama, Japan; ^3^Institute of Natural Medicine, University of Toyama, Toyama, Japan

**Keywords:** NAD, Warburg effect, Nampt, Naprt, CD38, PARP, sirtuin, FK866

## Abstract

Cancer cells have a unique energy metabolism for sustaining rapid proliferation. The preference for anaerobic glycolysis under normal oxygen conditions is a unique trait of cancer metabolism and is designated as the Warburg effect. Enhanced glycolysis also supports the generation of nucleotides, amino acids, lipids, and folic acid as the building blocks for cancer cell division. Nicotinamide adenine dinucleotide (NAD) is a co-enzyme that mediates redox reactions in a number of metabolic pathways, including glycolysis. Increased NAD levels enhance glycolysis and fuel cancer cells. In fact, nicotinamide phosphoribosyltransferase (Nampt), a rate-limiting enzyme for NAD synthesis in mammalian cells, is frequently amplified in several cancer cells. In addition, Nampt-specific inhibitors significantly deplete NAD levels and subsequently suppress cancer cell proliferation through inhibition of energy production pathways, such as glycolysis, tricarboxylic acid (TCA) cycle, and oxidative phosphorylation. NAD also serves as a substrate for poly(ADP-ribose) polymerase (PARP), sirtuin, and NAD gylycohydrolase (CD38 and CD157); thus, NAD regulates DNA repair, gene expression, and stress response through these enzymes. Thus, NAD metabolism is implicated in cancer pathogenesis beyond energy metabolism and considered a promising therapeutic target for cancer treatment. In this review, we present recent findings with respect to NAD metabolism and cancer pathogenesis. We also discuss the current and future perspectives regarding the therapeutics that target NAD metabolic pathways.

## Introduction

Cancer cells involve a unique energy metabolism that promotes their rapid cell proliferation (1). One of the characteristic features of cancer metabolism is the preference for anaerobic glycolysis under normal aerobic conditions, known as the Warburg effect (2, 3). Although aerobic glycolysis efficiently yields 32 molecules of ATP from one molecule of glucose, only 2 molecules of ATP are generated from one molecule of glucose during anaerobic glycolysis (4). This shift to anaerobic glycolysis under normal oxygen conditions in cancer cells appears inefficient; however, the advantages of anaerobic glycolysis include a faster rate of ATP production and reduced generation of reactive oxygen species (ROS) that are mainly produced by ETC during respiration (4). Therefore, cancer cells appear to utilize these advantages instead of focusing on the efficient ATP generation.

Another advantage of shifting energy metabolism to anaerobic glycolysis is the production of the building blocks required for cancer proliferation (5). Glucose 6-phosphate (G6P), the first product of glycolysis that is generated by hexokinase, bypasses glycolysis through the pentose phosphate pathway (PPP). 5-phosphoribose-1-pyrophosphate (PRPP) is an intermediate product of the PPP and is used in biosynthesis of purine and pyrimidine nucleotides. Thus, PPP is activated in cancer cells to maintain sufficient nucleotide pools to support DNA replication and RNA production (6). In some cancer cells, a large proportion of glucose is used in the serine *de novo* synthesis pathway, wherein 3-phosphoglycerate is used by D-3-phosphoglycerate dehydrogenase (PHGDH) (7). Serine metabolism is associated with the synthesis of ceramide, a component of the cellular membrane (8). Serine is also converted to glycine and connected to the folic acid and methionine metabolism (9, 10). Thus, the serine biosynthesis pathway is also considered critical for sustaining the growth of cancer cells.

Nicotinamide adenine dinucleotide (NAD) is a co-enzyme that mediates redox reactions in various metabolic pathways, including glycolysis, tricarboxylic acid (TCA) cycle, oxidative phosphorylation, and serine biosynthesis (11). Continuous replenishment of NAD promotes the proliferation and survival of fast-dividing cancer cells because elevated NAD levels enhance glycolysis via glyceraldehyde 3-phosphate dehydrogenase (GAPDH) and lactate dehydrogenase (LDH) that require NAD as a co-enzyme (12, 13). PHGDH, a rate-limiting enzyme of the serine biosynthesis pathway, also uses NAD as a co-enzyme, and the intracellular level of NAD is considered to be an important regulator for serine biosynthesis in cancer cells (9, 14). Furthermore, NAD serves as a substrate for poly(ADP-ribose) polymerase (PARP) and sirtuins (NAD-dependent deacetylases) and mediates poly-ADP-ribosylation and deacetylation, respectively. Thus, NAD metabolism is involved in energy metabolism, DNA repair, gene expression, and stress response via the action of these enzymes (15). Recently, several studies have indicated that NAD metabolism is involved in cancer development and progression and is considered a promising therapeutic target in cancer treatment. In this review, we summarize the roles of NAD metabolism in cancer pathogenesis. We also focus on the inhibitors of NAD-synthesis enzymes, and describe their implications in cancer treatment.

## NAD Synthesis and Consuming Pathways

**Figure 1:**
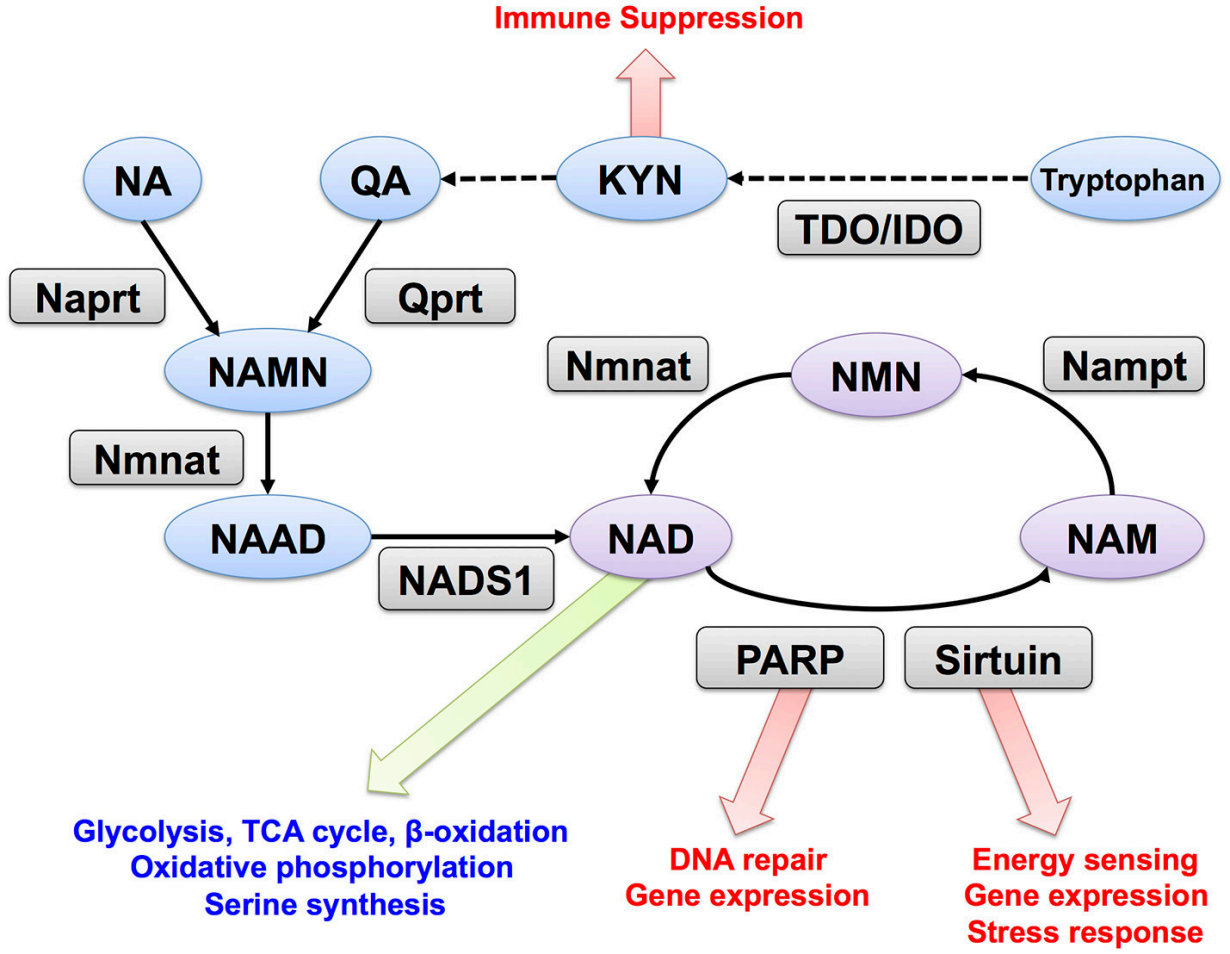
NAD metabolism and its downstream targets. Trp, tryptophan; KYN, kynurenine; NA, nicotinic acid; NAM, nicotinamide; QA, quinolinic acid; NMN, nicotinamide mononucleotide; NAMN, nicotinic acid mononucleotide; NAD, Nicotinamide adenine dinucleotide; NAAD, nicotinic acid adenine dinucleotide; Nampt, nicotinamide phosphoribosyltransferase; Nmnat, nicotinamide mononucleotide adenylyltransferase; Qprt, quinolinic acid phosphoribosyltransferase; Naprt, nicotinic acid phosphoribosyltransferase; NADS1, NAD synthetase; PARP, poly (ADP-ribose) polymerase. TDO, tryptophan 2,3-dioxygenase; IDO, indoleamine 2,3-dioxygenase.

NAD is synthesized through the *de novo*, Preiss-Handler, and salvage pathways from tryptophan, nicotinic acid (NA), and nicotinamide (NAM), respectively (Figure 1). In mammalian cells, two key enzymes, nicotinamide phosphoribosyltransferase (Nampt) and nicotinamide mononucleotide adenylyltransferase (Nmnat) regulate the salvage pathway that is considered critical in controlling intracellular NAD levels (11). Nampt converts NAM and PRPP to nicotinamide mononucleotide (NMN), and Nmnat generates NAD by transferring the adenylyl moiety from ATP to NMN (16, 17). In the Preiss-Handler pathway, nicotinate phosphoribosyltransferase (Naprt) generates nicotinic acid mononucleotide (NAMN) from NA and PRPP, and then Nmnat conjugates ATP to NAMN to generate NAD (18). In the *de novo* pathway, tryptophan is used as the source for NAD synthesis; further, tryptophan 2,3-dioxygenase (TDO) or indoleamine 2,3-dioxygenase (IDO) mediates the first step and acts as a rate-limiting enzyme in this pathway. In the salvage pathway, NAD degradation is coupled with NAM recycle (19). PARP and sirtuin use NAD as a substrate for ADP-ribosylation and deacetylation, respectively (20, 21). NAD glycohydrolases, CD38 and CD157, also consume NAD and generate ADP-ribose or cyclic-ADP-ribose (22, 23). All these enzymes generate NAM when they degrade NAD, and Nampt reuses NAM for NAD synthesis. In mammals, there are three Nmnat isozymes (Nmnat1–3) with different subcellular localizations and tissue distributions. Nmnat1, Nmnat2, and Nmnat3 are considered to be in the nucleus, Golgi apparatus, and mitochondria, respectively (24). Additionally, Nampt is primarily located in the cytoplasm, and its inhibition blocks glycolysis (13). Nmnat1 is reported to supply nuclear NAD and sustain the activity of PARP and sirtuin (25, 26). In mitochondria, NAD is utilized in TCA cycle, fatty acid oxidation, and oxidative phosphorylation (27). In fact, overexpression of Nmnat3 in mice increases mitochondrial NAD levels and enhances energy metabolism in mitochondria (28).

## Nampt Regulates Cancer Proliferation and Survival

Overexpression of Nampt is frequently observed in several types of malignant tumors, including, colorectal, ovarian, breast, gastric, thyroid, prostate cancers, gliomas, and malignant lymphomas (29–48). Increased NAD levels accompanied by Nampt overexpression sustain rapid cellular proliferation and promote cancer cell survival against anti-cancer cell reagents. In particular, elevated NAD levels boost glycolysis through glyceraldehyde 3-phosphate dehydrogenase (GAPDH) and lactate dehydrogenase (LDH) that require NAD as a co-enzyme and enhance anaerobic glycolysis (12, 13). A well-known oncogene, c-MYC was reported to regulate Nampt expression in cancer cells (49). c-MYC transcriptionally regulates the metabolic reprogramming of cancer cells by enhancing glucose uptake, glycolysis, and lactate production, the increase in Nampt expression by c-MYC may lead to the Warburg effects (50). Several microRNAs regulate Nampt levels and promote cancer cell proliferation. miR26b reportedly suppresses Nampt expression by binding to the 3'-UTR in the Nampt gene. In colorectal cancer cells, miR26b is down regulated, leading to the overexpression of Nampt (51). Similarly, decreased miR206 also induced Nampt expression in breast and pancreatic cancers. In contrast, the exogenous expression of miR-206 reduced the Nampt expression, resulting in a significantly reduced level of NAD and subsequent induction of cell death (44, 52). Other microRNAs, including miR374a, miR451a, and miR568 reportedly regulate Nampt levels in cancer cells (51, 53, 54).

Nampt was originally identified as a cytokine named pre-B-cell colony-enhancing factor (also known as a visfatin), and exists in serum as a secreted form (55, 56). An extracellular form of Nampt (eNampt) is released from multiple types of cells, including mature adipocytes, hepatocytes, myocytes, neurons, and immune cells (57–62). Moreover, eNampt was released from cancer cells, and the eNampt level is reportedly associated with the progression of certain types of cancers (63–68). eNampt induces inflammatory cytokines in the leukocytes, macrophages, gut epithelial cells, and endothelial cells (64, 69, 70) and promotes cancer-related inflammation. Furthermore, eNampt has been reported to polarize the monocytes toward tumor-supporting M2-macrophages. eNampt increases the expression of immunosuppressive molecules, such as IDO, CCL-18, and IL-10, as well as tumor-promoting cytokines, such as IL-1β and IL-6 (64, 69). eNampt also acts as a pro-angiogenic factor by promoting the expressions of VEGF and MMP2/9 through the MAPK and PI3K/Akt signaling pathways (71–73). Overexpression of Nampt also promotes epithelial-to-mesenchymal transition (EMT) by increasing eNampt, and activates the TGFβ signaling pathway (74). In addition, Nampt inhibition can kill the gastric cancer cell lines with EMT-associated gene expression signatures (75). These results suggest that specific neutralization of eNampt can be used as a potential anti-cancer strategy.

## Targeting Cancer Metabolism With Nampt Inhibitors

A number of studies have demonstrated that the overexpression of Nampt contributes to cancer pathogenesis and development; therefore, Nampt is considered as a potential therapeutic target for cancers. Accordingly, several kinds of Nampt inhibitors have been developed (Table 1). FK866 (also known as APO866) is the first chemical compound discovered to be a highly specific inhibitor of Nampt (76). It inhibits the enzymatic activity of Nampt non-competitively by binding to an allosteric regulatory site (76). The treatment of cultured cancer cells by FK866 significantly reduces intracellular NAD levels followed by the inhibition of the glycolysis pathway catalyzed by GAPDH (13, 93). Thus, FK866 suppresses cancer cell growth by targeting energy metabolism, particularly glycolysis (94). Another research has also shown that FK866 kills blood cancer cells by inducing apoptosis and/or caspase-independent autophagic cell death (95).

**Table 1:** Small molecules targeting NAD synthesis enzymes.

**Target enzymes**	**Compound name**	**Property**	**Implication in cancer treatment**	**References**
Nampt	FK866 (APO866)	Non-competitive inhibitor	Phase I study in advanced solid tumors: Thrombocytopenia was the dose limiting toxicity. No objective tumor responses were observed	(76, 77)
	GMX-1778 (CHS-828)	Pyridyl cyanoguanidine	Phase I study in advanced resistant solid tumors: Gastrointestinal toxicitiy and thrombocytopenia were observed. No objective tumor responses were observed	(78–81)
	GMX-1777	Pro-drug of GMX-1778	Phase I study in advanced malignancies: Thrombocytopenia and gastrointestinal hemorrhage was the dose limiting toxicity. No objective tumor responses were observed	(82, 81)
	STF-31	Dual inhibitor of Nampt and Glut1	Deplete NAD levels, and inhibit glucose uptake and glycolysis	(83, 84)
	STF-118804	Competitive inhibitor	Induce apoptosis antecedent cell cycle arrest	(85, 86)
	GNE-617	Competitive inhibitor	Deplete NAD and ATP levels, and promote cell death	(87, 88)
	GNE-618	Competitive inhibitor	Deplete NAD and ATP levels, and promote cell death	(87, 89)
	LSN3154567	Competitive inhibitor	Alone or coadministered with NA exhibits a potent antitumor activity in tumor xenograft models. The retinopathy associated with LSN3154567 could be mitigated with NA coadministration	(90)
	KPT-9274	Dual inhibitor of Nampt and PAK4	KPT-9274 decreases G2/M transit and causes apoptosis	(91)
Nmnat2	VAD	NAD analog inhibiting both Nampt and Nmnat2	Induce NAD depletion, glycolytic block, energy failure, and necrotic death.	(92)

Other Nampt-specific inhibitors, such as GMX1777 and GMX1778, have exhibited anti-cancer effects similar to those of FK866 (78). GMX1777 is a cyanoguanidine compound and a pro-drug of GMX1778 (also known as CHS-828) (82). It is rapidly converted into the active form GMX1778, a competitive inhibitor of Nampt (79). The combination of GMX1777 and radiotherapy has demonstrated therapeutic efficacy in a mouse model of head and neck cancer (96). STF-31 was originally identified as a compound that selectively kills renal cell carcinoma with a loss of the von Hippel-Lindau (VHL) tumor suppressor gene (83). STF-31 inhibits glucose uptake and the Warburg effect by targeting Glut1 (83). A more recent study has demonstrated that recurrent NAMPT-H191R mutations confer resistance to STF-31 treatment in cancer cell lines and that STF-31 also exerts an additional inhibitory effect against Nampt (84). Thus, these dual functions are beneficial for countering tumors with a high glucose consumption rate.

Although Nampt inhibitors are promising candidates for preventing tumor cell growth, Nampt is also essential for normal cells. In fact, whole body deletion of Nampt results in embryonic lethality, and muscle-specific Nampt deficient mice exhibit progressive muscle degeneration (57, 97). Moreover, retina-specific Nampt deficient mice exhibit severe vision loss (98). Therefore, it is important to consider these adverse side effects while using Nampt inhibitors in clinical settings.

## Naprt and Cancer Metabolism

In contrast to the cases with respect to Nampt, several types of cancer cells reportedly lack in Naprt expression (99, 100). These cells cannot utilize NA for NAD synthesis through Naprt; hence, NAD synthesis solely depends on Nampt. Therefore, the efficacy of FK866 treatment is higher than that in cancer cells that express Naprt. In fact, the DNA hypermethylation of the promoter region of *Naprt* is frequently observed in certain types of cancers, and this could be used as an effective marker for treatment with FK866 (101). Normal cells usually express both Naprt and Nampt; therefore, co-administration of Nampt inhibitor and NA can preserve NAD levels through NA-Naprt pathway in normal cells. On the other hand, Nampt inhibitor effectively decreases NAD levels in cancer cells lacking Naprt, and subsequent ATP depletion due to impaired glycolysis can selectively kill cancer cells (100). Similarly, selective inhibition of Nampt by GMX1778 blocks NAD production and induces cell death in Naprt-deficient tumors, while NA co-supplementation can rescue NAD depletion in Naprt-positive normal cells, bypassing the NA-Naprt pathway (79). Thus, the Naprt status in tumor cells is a critical indicator in the treatment of cancers using Nampt inhibitors.

Isocitrate dehydrogenase (IDH) mutations in glioma cell lines are reported to decrease the expression of Naprt via increased DNA and histone methylation (102). Mutations in the IDH gene are frequently observed in patients with gliomas and acute myeloid leukemia (AML). Mutated IDH aberrantly generates 2-hydroxyglutarate (2-HG), which inhibits DNA and histone demethylases and promotes the hyper methylation of DNA histones (103). In IDH-mutated cells, NAD levels are significantly decreased due to decreased Naprt, making them sensitive to NAD depletion by Nampt inhibitors, such as FK866 and GMX1778 (102, 104). Decreased NAD levels in FK866-treated IDH-mutant cells suppress the flux in TCA cycle and cause reduction in ATP production, which activates the intracellular energy sensor AMPK, triggers autophagy, and finally leads to both autophagic cell death (102). In fact, 3-methyladenine, an inhibitor of autophagy, partially rescues this cytotoxicity (102, 105). Thus, IDH mutation in tumors, including gliomas and AML, is considered a good indicator of the suitability of Nampt inhibitor therapy.

It is noteworthy that aberrant expression of unconventional prefoldin RPB5 interactor (URI) in hepatocytes causes NAD depletion, ultimately leading to spontaneous liver tumor formation (106). URI downregulates the gene expression of enzymes in the kynurenine pathway through aryl hydrocarbon receptor and estrogen receptors. Depleted NAD levels subsequently promote DNA damage and result in the onset of hepatocellular carcinoma. Notably, nicotinamide riboside (NR) supplementation restores the NAD levels in URI-expressing hepatocytes and prevents DNA damage and tumor formation. In human hepatocellular carcinoma patients, URI expression is negatively correlated with that of the enzymes in the kynurenine pathway and is associated with a poor prognosis (106).

## Nmnat as a New Candidate for Targeting Cancer Metabolism

Nmnat inhibitors are expected to exhibit anti-cancer effects via the inhibition of the NAD synthesis pathway (107). Nmnat2 is amplified in colorectal cancer and exhibits a positive correlation with p53 expression. Furthermore, it is reported that the expression of Nmnat2 under DNA damage was induced by p53 (108). Reportedly, SIRT3 activates Nmnat2 through deacetylation and increased NAD levels in non-small cell lung-cancer cells (109). SIRT3-Nmnat2-NAD axis dictates the energy metabolism and cellular proliferation, and disrupting the interaction between SIRT3 and Nmnat2 promotes apoptosis. These results imply that Nmnat2 can be a therapeutic target (109). A recent study has reported that vacor adenine dinucleotide (VAD) inhibits Nmnat2 and Nmapt (92). It is noteworthy that the administration of vacor to Nmnat2-expressing cancer cell leads to VAD formation and depletes NAD pools (92). Furthermore, vacor leads to growth suppression and necrotic cell death via blockage of glycolysis in Nmnat2-positive cancer cells; however, it has no effect on Nmnat2-lacking cells. Overexpression of Nmnat2 in colorectal cancer was reported to enhance cell death induced by tiazofurin, an analog of NAD. Nmnat2 enzymatically converts tiazofurin to thiazole-4-carboxamide adenine dinucleotide, a potent inhibitor of inosine 5'-monophosphate dehydrogenase, required for guanylate synthesis in colorectal cancers (110). Taken together, amplification of Nmnat2 in cancer cells could be a unique target for these compounds.

## NAD Consuming Enzymes and Cancer Development

Intracellular NAD levels also influence the activities of the NAD consuming enzymes, PARP and sirtuin, and regulate cancer initiation and progression. NAD serves as a substrate for PARP that plays crucial roles in DNA repair, chromatin modification, cell transformation, and cell death (20). In particular, DNA repair is targeted by several cancer therapies, including chemotherapy and radiation therapy. Recently, PARP inhibitors have been identified as promising therapeutic agents against breast and ovarian cancers with BRCA mutations. Because BRCA mutations impair the homologous recombination of DNA double-strand breaks (DSBs) in cancer cells, PARP inhibitors increase DNA single-strand breaks (SSBs) and lead to synthetic lethality. Poly ADP-ribosylation during the DNA repair process consumes a large amount of NAD; limiting NAD synthesis may also induce cancer cell death by inhibiting the PARP activity (20). In fact, the combination of a PARP inhibitor and FK866 induced synthetic lethality in triple-negative breast cancer (111). It has also been reported that a combination of PARP inhibitors and β-lapachone, a natural quinone, exhibits synergistic anti-tumor activity against NAD(P)H:quinone oxidoreductase 1 (NQO1)-overexpressing cancers, such as non-small-cell lung cancer, pancreatic cancer, and breast cancer (112). NQO1 is an enzyme that couples NAD(P) oxidation with a reduction of quinone to hydroquinone, and β-lapachone is converted to an unstable hydroquinone that spontaneously generates ROS and induces DNA damages (113). However, β-lapachone also promotes hyper activation of PARP and damages normal cells by depleting NAD and ATP levels (112, 114). Thus, the co-administration of β-lapachone and PARP inhibitors to cancer cells generates considerable amount of hydrogen peroxide without affecting the NAD pools that constantly refuel NQO1 redox cycling and induce massive DNA damage (112). Therefore, the combination of PARP inhibitors and β-lapachone blocks DNA repair and induces tumor-selective apoptosis in NQO1-overexpressing cancers.

The NAD-dependent deacetylases (sirtuins) affect various processes involved in the suppression of tumor initiation and maintenance. Thus, a disturbance in the NAD levels could influence sirtuin-mediated suppression of tumor formation and growth. In fact, dysregulated c-Myc initiates a positive feedback loop between Nampt, the SIRT1 inhibitor DBC1, and SIRT1, that contributes to tumor development and maintenance (49). Recently, it has been reported that Nampt expression is correlated to a large number of cancer-initiating cells in colon cancer patients, and transcription meta-analysis has revealed that Nampt regulates the pathways involved in the maintenance of cancer stemness in colon cancers in a SIRT1- and PARP1-dependent manner (30).

More than 90% of multiple myeloma cells from patients have surface expression of CD38 (115). Daratumumab, an anti-CD38 monoclonal antibody, has been approved as a therapy for multiple myeloma. Daratumumab strongly induces antibody-dependent cellular cytotoxicity and kills myeloma cells. However, the pathological significance of CD38 expression in multiple myeloma cells remains unclear. CD38 reportedly regulates intracellular NAD levels, and CD38 deficiency in mice increases the NAD levels in multiple tissues (116). A recent study has indicated that CD38 expression inversely correlates with the progression of prostate cancer (117, 118). Thus, the inhibition of the enzymatic activity of intracellular CD38 may have adverse effects on tumor proliferation. In myeloma cells, CD38 is overexpressed ectopically, and it is unclear whether extracellular NAD is involved in tumor progression. CD157/BST1 is a paralog of CD38 and shares similar NADase activity. In ovarian cancer, CD157 is often overexpressed and contributes to cell migration and peritoneal invasion (119, 120). Thus, the inhibition of CD157 may also contribute to cancer therapy.

## Conclusion

The Warburg effect was discovered more than half a century previously, and the strategy targeting cancer metabolism is regaining popularity. NAD is an important co-factor that mediates a number of metabolic pathways, including glycolysis. In fact, Nampt is overexpressed in several types of malignant tumors and is considered a potential therapeutic target. Various Nampt-specific inhibitors have been developed, and they exhibit effective responses to cancer cells in pre-clinical models (76, 78, 82, 83, 85–91). Although Nampt inhibitor is a promising anti-cancer reagent, several challenges remain with respect to the application in human patients. Most importantly, Nampt is also essential for normal cells, and complete deletion of Nampt in mice has shown to have adverse effects in the retina, bone marrow, liver, and blood platelets. In fact, clinical trials using Nampt inhibitors have encountered hematological toxicities, and their clinical development has been stagnated (77, 80, 81, 121). Therefore, it is important to select patients with therapeutic adaptation to Nampt inhibitor. Thus, the status of Naprt expression is considered a good indicator of the suitability of Nampt inhibitor therapy, and the co-administration of Nampt inhibitor and NA, a substrate for Naprt, is a promising strategy to decrease the toxicity in normal cells. IDH mutation increases Naprt expression in glioma cells, and patients with this mutation may be suitable for Nampt inhibition therapy (102, 105). A recent study has demonstrated that treatment with Nampt inhibitor-containing microparticle successfully extended the survival of mice with IDH-mutated gliomas. Further, the research group has developed a method to determine the presence of mutation in glioma cells using surgical specimens within 30 min. If the test is positive, patients can be treated locally by applying Nampt inhibitor-containing microparticle to the primary lesion during operation (104). Thus, this procedure is considered ideal to avoid adverse side effects and maximize anti-tumor effect to suitable subjects for Nampt inhibitor therapy. Another option is to induce a synthetic lethality using Nampt inhibitors and other anti-cancer drugs. It has been known that the repair process of SSB DNA damage consumes a large amount of NAD through PARP-mediated poly ADP-ribosylation. Thus, the combination with DNA damage inducing reagent and Nampt inhibitor may synergistically induce cell death in cancer cells.

## Author Contributions

KY, KO, KH, and TN wrote the manuscript. TN and KY revised and edited manuscript.

### Conflict of Interest Statement

The authors declare that the research was conducted in the absence of any commercial or financial relationships that could be construed as a potential conflict of interest.
